# Sequestering HMGB1 via DNA-Conjugated Beads Ameliorates Murine Colitis

**DOI:** 10.1371/journal.pone.0103992

**Published:** 2014-08-15

**Authors:** Zhongliang Ju, Sangeeta S. Chavan, Daniel J. Antoine, Meghan Dancho, Teá Tsaava, Jianhua Li, Ben Lu, Yaakov A. Levine, Andrew Stiegler, Yehuda Tamari, Yousef Al-Abed, Jesse Roth, Kevin J. Tracey, Huan Yang

**Affiliations:** 1 Laboratory of Biomedical Science, The Feinstein Institute for Medical Research, Manhasset, New York, United States of America; 2 MRC Centre for Drug Safety Science, Department of Molecular and Clinical Pharmacology, Institute of Translational Medicine, University of Liverpool, Liverpool, United Kingdom; 3 SetPoint Medical Corporation, Valencia, California, United States of America; 4 Circulatory Technology Inc., Oyster Bay, New York, United States of America; 5 Medicinal Chemistry, The Feinstein Institute for Medical Research, Manhasset, New York, United States of America; University of Chicago, United States of America

## Abstract

Inflammatory bowel disease (IBD) is chronic inflammation of the gastrointestinal tract that affects millions of people worldwide. Although the etiology of IBD is not clear, it is known that products from stressed cells and enteric microbes promote intestinal inflammation. High mobility group box 1 (HMGB1), originally identified as a nuclear DNA binding protein, is a cytokine-like protein mediator implicated in infection, sterile injury, autoimmune disease, and IBD. Elevated levels of HMGB1 have been detected in inflamed human intestinal tissues and in feces of IBD patients and mouse models of colitis. Neutralizing HMGB1 activity by administration of anti-HMGB1 antibodies or HMGB1-specific antagonist improves clinical outcomes in animal models of colitis. Since HMGB1 binds to DNA with high affinity, here we developed a novel strategy to sequester HMGB1 using DNA immobilized on sepharose beads. Screening of DNA-bead constructs revealed that B2 beads, one linear form of DNA conjugated beads, bind HMGB1 with high affinity, capture HMGB1 *ex vivo* from endotoxin-stimulated RAW 264.7 cell supernatant and from feces of mice with colitis. Oral administration of B2 DNA beads significantly improved body weight, reduced colon injury, and suppressed colonic and circulating cytokine levels in mice with spontaneous colitis (IL-10 knockout) and with dextran sulfate sodium-induced colitis. Thus, DNA beads reduce inflammation by sequestering HMGB1 and may have therapeutic potential for the treatment of IBD.

## Introduction

Inflammatory Bowel Disease (IBD), which includes ulcerative colitis and Crohn's disease, is one of the five most prevalent gastrointestinal diseases, with an annual cost of more than $1.7 billion in the United States [1]–[3]. The etiology of IBD remains unclear, but it is associated with a considerable reduction in quality of life and significant morbidity [4]–[7]. Despite significant progress in the management of the disease, curative treatment options are not yet available. Current therapeutics targeting excessive cytokine production or using immune-suppressive regimens have had limited success [3], [4], [8].

High mobility group box 1 (HMGB1) is a ubiquitous nuclear protein involved in nucleosome stabilization, gene transcription and neurite outgrowth [9]. During infection or injury, activated immune cells and damaged cells release HMGB1 into the extracellular space, where HMGB1 functions as a pro-inflammatory mediator and contributes to the pathogenesis of inflammatory diseases [10]–[12]. HMGB1 has recently been implicated in the pathogenesis of IBD. In IBD patients and mice with colitis, HMGB1 is secreted by inflamed intestinal tissues and present at high levels in the feces [13], [14]. The large quantities of HMGB1 in the gastrointestinal tract mediate inflammation and gastrointestinal barrier failure [15], [16]. Neutralizing HMGB1 activity by administration of anti-HMGB1 antibodies or ethyl pyruvate attenuates colon injury, reduces weight loss and improves colon scores in animal models of colitis [13], [14], [17], [18]. Together these findings suggest that HMGB1 could be an important therapeutic target in IBD.

Recent extensive studies have demonstrated that redox state of HMB1 determines both intracellular and extracellular functions of HMGB1. Importantly, HMGB1 contains three cysteines (C23, C45 and C106), each of which is susceptible to redox modification [19].The redox state of these cysteine residues determines the biological activity of extracellular HMGB1 [19]–[21]. Cytokine-stimulating HMGB1 has C23 and C45 in a disulfide linkage and C106 in its reduced form with a thiol side chain and has been recently re-named as disulfide HMGB1. When all cysteine residues are reduced, HMGB1 acts as a chemotactic mediator, this molecular form has been recently named fully reduced HMGB1 [22]. When all cysteine residues are terminally oxidized to the sulphonate, HMGB1 has no cytokine-stimulating or chemotactic activity (sulfonyl HMGB1). Other post-translational modifications such as acetylation and phosphorylation have been implicated in the regulation of HMGB1 release. HMGB1 contains two nuclear localization sequences (NLS), and lysine residues in NLS regions are susceptible to acetylation modification. It has been shown that hyperacetylation of HMGB1 at the NLS results in nuclear exclusion and subsequent HMGB1 release [23]–[25].

HMGB1 exerts strong binding to DNA including linear, bends, bulges and four-way junctions [9], [26]–[28]. The DNA-binding property of HMGB1 has been utilized to neutralize HMGB1 cytokine activity, reduce immune responses and ameliorate the severity of diseases in animal models of inflammation associated with elevated levels of HMGB1 [29], [30]. Here we developed a novel strategy to sequester HMGB1 using DNA immobilized on sepharose beads (45–165 µm, average size 90 µm). These DNA beads bind HMGB1 with high affinity, capture HMGB1 *ex vivo* from activated RAW 264.7 cell supernatants and from feces of colitis mice. When administered orally, these DNA beads improves body weight, reduces colon injury and suppresses colonic and circulating cytokine levels in mice with spontaneous colitis (IL-10 knockout mice) or with dextran sulfate sodium-induced colitis.

## Materials and Methods

### Materials

CNBr-activated sepharose 4 fast flow resin and CM5 dextran sensor chip were from GE healthcare (Piscataway, NJ). Lipopolysaccharide (LPS, *E. Coli*. 0111:B4), Recombinant human histone H3, triton X-100 and protease inhibitors were purchased from Sigma (St. Louis, MO). Dextran sulfate sodium (MW = 36,000–50,000 Da) was purchased from MP Biomedicals (Solon, OH). RAW 264.7 (TIB-71), HELA (CCL-2) and Caco-2 (HTB-37) cells were obtained from American type culture collection (Rockville, MD). Human TNF and anti-human TNF antibody were purchased from R & D system Inc. (Minneapolis, MN). The lactate dehydrogenase (LDH) cytotoxicity kit was from Takara Inc., (Otsu, Shiga, Japan). HMGB1 ELISA kit was from IBL international (Hamberg, Germany). QIAzol lysis reagent was from Qiagen (Valencia, CA). One step RT-PCR kit was obtained from Eurogentec (Fremont, CA). Poly I∶C was from InvivoGen (San Diego, CA). Light Cycler 480 instrument was purchased from Roche Diagnostics (Munich, Germany). RNase free DNase I and anti-histone H3 antibody were purchased from Thermo Scientific (Rockford, IL). Superscript III First-strand Synthesis System for cDNA synthesis was purchased from Invitrogen (Grand Island, NY). Microcon centrifugal filters were from Millipore (Billerica, MA).

### Cell culture

Cells (RAW 264.7, Hela and Caco 2) were cultured in DMEM supplemented with 10% or 20% (for Caco2 cells) of fetal bovine serum, 100 U/ml penicillin and 100 µg/ml streptomycin. Cells were used at 90% confluence and treatment was carried out in serum-free Opti-MEM I medium (Life Technologies, Carlsbad, CA). Human primary monocytes were purified by density gradient centrifugation through Ficoll from blood donated by healthy volunteers to the Long Island Blood Bank (New York Blood Center, Melville, NY) as previously described [11].

### Generation of oligos

We selected four different DNA constructs that were reported to have high binding affinity to HMGB1, including two non-immunogenic linear DNA (S1 and S2 [30], Kd to HMGB1 = 5 nM for both), 4-way junction DNA (S3, Kd to HMGB1 = 1 nM, [28]) and a kinked duplex DNA (S4, Kd to HMGB1 = 22 nM, [29]) (Table 1). S1 refers to 12–20.1; S2 refers to 12–20.2; S3 refers to 12-4WJ.1; S4 refers to 12-DP.1.

**Table 1:** Design of DNA oligos.

DNA	Oligos	Sequence 5′ to 3′	Affinity (Kd)
S1	1	AmC6AGCATGAGGTTCCTGATGCT	5 nM
S2	2	AmC6TGGATGAGCTTCCTGATGTC	5 nM
S3	3	AmC6CCCTATAACCCCTGCATTGAATTCCAGTCTGATAA	1 nM [28]
	4	GTAGTCGTGATAGGTGCAGGGGTTATAGGG	
	5	AACAGTAGCTCTTATTCGAGCTCGCGCCCTATCACGACTA	
	6	TTTATCAGACTGGAATTCAAGCGCGAGCTCGAATAAGAGCTACTGT	
S4	7	AmC6CTTGCATTGAAATTTCTTTCC	22 nM [29]
	8	GAACGTAACAAAGAAAGG	

Four DNA molecules (S1–S4) were selected for this study [28]–[30]. An amino linker (Am) was added to 5′ end of sequences 1, 2, 3 and 7. Biacore T200 was used for analysis of binding between HMGB1 (coated on the chip) and DNA S1 and S2 (0–10 µM, Methods). HMGB1 binds in a similar manner to both S1 and S2, in an HMGB1 concentration-dependent manner and with high affinity (Kd = 5 nM). Data are representative of two repeats.

All oligonucleotides (sequences 1–8, Table 1) were made by Genemed Synthesis, Inc. (San Antonio, TX). A C6 amino group linker [NH2(CH2)_6_O-P(O)2] was conjugated to the 5′ of the following oligos: sequences 1, 2, 3 and 7. To prevent DNase degradation, oligos were synthesized with phosphorothioate for two linear forms of DNA S1 and S2 and with phosphodiester backbone for DNA forms of S4 and S3 throughout the sequences. The oligos were selected and synthesized according to previous publications [28]–[30].

### Preparation of DNA beads

Conjugation of DNA to CNBr sepharose beads was carried out as described by Chockalingam et al [31]. The coupling efficiency of the samples was calculated by measuring the pre- and post- coupling OD_260_ and the percentage of the DNA immobilized to sepharose beads after the coupling reaction. Control beads were prepared using the same procedure but without adding DNA. The beads coated with S1, S2, S3 and S4 were referred as B1, B2, B3 and B4 beads respectively. Approximately 8 nmol of S1 and S2 DNA (48 µg), 4 nmol of S3 and S4 DNA (180 µg) were bound to each ml of drained beads.

### Binding ratio of HMGB1 to DNA beads

Each of the three DNA-beads showed maximum binding at 2 µg HMGB1 which corresponds to 76 pmols of HMGB1. The concentration of DNA in beads is approximately 8 µM for B1 and B2, and 4 µM for B3. Given that 76 pmoles of HMGB1 bound 5 µl of DNA beads, it was calculated that the binding ratio of HMGB1 to B1 and B2 beads is 2∶1 (76 pmoles HMGB1 per 40 pmoles of DNA = 2) and for B3 is 4∶1 (76 pmoles HMGB1 per 20 pmoles of DNA = 4).

### Binding of HMGB1 and DNA beads

The binding ability and affinity of the DNA beads to HMGB1 was tested using a depletion approach. A fixed amount of drained DNA beads B1, B2, B3 and B4 (5 µl) were incubated with HMGB1 at 0, 0.01, 0.1, 0.2, 0.5, 1, 2 and 3 µg in a total volume of 50 µl at room temperature for two hours with rotation. Alternatively fixed amount of HMGB1 (2 µg) was incubated with increasing amounts of DNA beads (0, 0.2, 1.5, 5, and 15 µl drained DNA beads) in a total volume to 100 µl for 2 hours at room temperature with rotation. The bound and free HMGB1 was analyzed by western blot [32]. To determine binding kinetics, DNA beads (20 µl) were incubated with 500 ng of HMGB1 in 50 µl PBS for 0, 15, 30, 60, 120, 240 or 960 minutes at room temperature and the amount of HMGB1 bound to the beads was measured by western blot.

### FAM-labeled B2 DNA-beads

Carboxyl terminal FAM-labeled S2 conjugated to sepharose beads were made by Genemed Synthesis Inc (San Antonio, TX). Fecal extract from colitis mice (300 µl) was incubated with FAM-labeled B2 beads (50 µl) at 37°C. After 2 hours incubation, beads were washed five times with PBS and re-suspended as 50% slurry. The fluorescence intensity in both supernatants and beads (before and after the incubation) was measured using a microplate spectrophotometer (Winooski, VT) at an excitation of 494 nm.

### Preparation of HMGB1 proteins

Recombinant HMGB1 was expressed in *E. coli* and purified to homogeneity, and endotoxin was extracted with triton X-114 [10], [33]. Redox-modified HMGB1 proteins were made as previously described [19], [34]. The LPS content in HMGB1 protein preparations was verified to be less than 10 pg/mg protein using Chromogenic Limulus Amebocyte Lysate Assay (Lonza Inc., Walkersville, MD).

### Cytokine measurements

TNF and IL-6 released in the cell culture supernatants were measured by ELISA as per manufacturer's instructions (R & D System Inc., Minneapolis, MN). HMGB1 levels were measured by western blot or ELISA [11].

### Surface plasmon resonance analysis

Binding of HMGB1 to DNA molecules was analyzed by surface plasmon resonance analysis using the BIAcore T200 instrument (BIAcore Inc, NJ). Recombinant HMGB1 was immobilized on a CM5 dextran sensor chip, and different concentrations of DNA (S1 or S2, 0–10 µM) were used as analyte. The association of analyte and ligand was recorded using surface plasmon resonance and the results were analyzed using BIAeval 3.2 software.

### Stability of DNA beads

To determine stability of DNA beads in acidic conditions, DNA beads (B1, B2 or B3, 20 µl) were incubated with 500 ng of HMGB1 in PBS (pH 7.0) or HCl (pH 1 or 2) for an hour at room temperature. In addition, susceptibility to DNase I-mediated digestion was evaluated by pretreating DNA beads B2 with a biological concentration of DNase I (40 ng/ml) for 30 minutes at 37°C [35] prior to incubation with of HMGB1 (0, 0.01, 0.1, 0.2, 0.5, 1, 2, 3 µg/5 µl beads) for 1 hour at room temperature. After washing beads with PBS, HMGB1 bound to the beads was eluted and measured by western blot.

### Immunogenicity of DNA oligos and DNA beads

Human primary macrophages were stimulated with HMGB1 (1 µg/ml) or LPS (2 ng/ml) in the presence or absence of S1, S2 or S3 DNA (1 µM or as indicated) at 37°C for 16 hours. TNF levels were measured in the supernatant. In separate experiments, murine macrophage-like RAW 264.7 cells were incubated with LPS (2 ng/ml), poly I∶C (1 µM), S1 or S2 (1 µM), or B1 and B2 beads (100 µl) for 24 hours at 37°C. Total RNA was extracted using QIAzol Lysis Reagent, and treated with DNase I to remove any contaminated genomic DNA. cDNA was synthesized by using a Superscript III First-strand Synthesis kit. The levels of IFN-β mRNA were analyzed by quantitative PCR using a Roche Light Cycler 480. Primers sequences used in IFN-β PCR amplification were as follows: forward 5′GCATCACCTGCTATGGGATT3′ and reverse 5′TACCCCAGGTTCTGGCTTTA3′. The PCR amplification was performed by denaturing the DNA at 95°C for 10 min, followed by 45 cycles of denaturing at 95°C for 10 seconds, annealing at 60°C for 30 seconds, and an extension at 72°C for 60 seconds. Relative mRNA expression was normalized to the expression of HPRT housekeeping gene [36].

### Cytotoxicity of DNA beads

Human cervical cancer cell line Hela and human epithelial colorectal adenocarcinoma cell line Caco-2 were incubated with increasing concentrations of control beads or with B2 DNA-beads for various time periods at 37°C. Cell death was determined by analyzing levels of lactate dehydrogenase (LDH) in the supernatant using LDH cytotoxicity kit. Triton X-100 (2%) was used as a positive control.

### Capture of HMGB1 using DNA beads from biological samples *in vitro*

RAW 264.7 cells in 6-well plates were stimulated with LPS (100 ng/ml) for 16 hours to induce the release of HMGB1. The cell supernatant was concentrated 10× using Microcon centrifugal filters, and incubated with B1, B2 or control beads (100 µl supernatant/20 µl beads) at room temperature for an hour with gentle rotation. Bound and free HMGB1 levels were measured by western blot.

### Animals

Female IL-10 knockout (KO) mice on C57BL/6J background (12 weeks old, stock #002251) were purchased from JAX laboratory (Bar Harbor, ME). Female and male C57BL/6J or BALB/c (8–12 weeks old) mice were purchased from Taconic laboratory (Germantown, NY). Mice were housed in the Feinstein Institute for Medical Research Animal Facility under standard temperature, and light and dark cycle. All animal procedures were carried out in strict accordance with the recommendations in the Guide for the Care and Use of Laboratory Animals of the National Institutes of Health. The protocol was approved by the Institutional Animal Care and Use Committee of the Feinstein Institute for Medical Research at North Shore-LIJ Health System (Animal Welfare Assurance number A-3168-01). Acute colitis was induced by feeding female BALB/c mice with 4% DSS dissolved in drinking water *ad libitum* for five days, followed by normal water for three days. Animals were euthanized after overnight fasting on day 9 [13], [14], [17]. For chronic colitis model, IL-10 KO mice were monitored for development of colitis.

### Colon tissue explants cultures

For *ex vivo* culture of the colon, full length colons from normal C57B/L6 mice were isolated, both ends tied and incubated with HMGB1 or PBS (in 0.5 ml volume) at room temperature with gentle shaking. After 1 hour incubation, LDH content was measured in the colonic suspension. In separate experiments, full-length colons were isolated from individual DSS-colitis mice, tied at both the ends and incubated with 0.5 ml of 50% B1 or B2 beads at room temperature for two hours with gentle shaking. The beads were harvested, washed thoroughly with PBS and analyzed by western blot.

### *Ex vivo* capture of HMGB1 from serum samples of septic mice, and fecal samples from colitis mice

Male, C57 BL/6 mice (8–12 weeks of age) were subjected to a cecal ligation and puncture (CLP) procedure [11]. Serum samples were collected from CLP and control mice after 48 hours, and incubated with 20 µl of B1, B2 or control beads at room temperature for an hour. Bound and free HMGB1 levels were analyzed on beads and supernatant using western blot and ELISA respectively. In separate experiments, fecal samples from DSS-colitis colons were gently flushed out with cold PBS containing 1× protease inhibitor, and the suspension was rotated for an hour at room temperature in the presence of gentamycin and imipenum. After centrifugation to remove fecal debris, fecal extract containing 40 µg of total protein was incubated with B1, B2 or control beads at room temperature for an hour. Bound HMGB1 on beads was analyzed after washing the beads with PBS by western blot.

### Analysis of HMGB1 isoforms by liquid chromatography and mass spectrometric analysis (LC-MS/MS)

HMGB1 from colitis fecal samples was captured using DNA beads, and eluted using 8M urea. HMGB1 in the eluted samples were confirmed by western blot analysis. Cell lysate from intestinal epithelial (Caco 2) cells was used as control for intracellular HMGB1. Analysis of LC-MS/MS was performed at Liverpool, UK. Samples were subjected to digestion by trypsin (Promega, Madison, WI) or endopeptidase GluC (New England Bio Labs., Ipswich, MA) according to the manufacturer's instructions. Characterization of cysteine bonds and absolute quantification of acetylated HMGB1 was determined as described previously [24], [25].

### Oral administration of DNA beads in colitis mice

B2, B4 or control beads (300 µl of 50% slurry) were orally administered to female BALB/c (10–12 weeks old) on days 0, 1, 2, 4 and 6 after DSS administration or to IL-10 KO mice starting at 12 weeks of age (when they spontaneously develop IBD). BALB/c mice were euthanized on day 8 after DSS, and IL-10 KO mice received B2 beads once every other day for a total of six weeks. Body weight was monitored daily (for BALB/c mice) or every other day (for IL-10 KO mice). At the time of euthanization, full length colon was collected, and its weight measured. Blood, feces and colon tissues were harvested for analysis.

### Histology

Colon tissues were fixed in 10% formalin and embedded in paraffin. Five-µm hematoxylin and eosin (H&E) stained sections were prepared by AML laboratory (Baltimore, MD). Colitis scores were determined for each high power view (magnification 40×) and 10 fields were viewed for each sample. The histological scoring system to quantify the degree of colitis was described previously and was evaluated in a blinded fashion [37]. The score ranged from 0 to 14 and was defined as follows. The inflammation severity was scored as 0–3 (0, no sign of inflammation; 1, mild inflammation; 2, moderate inflammation; 3, severe inflammation). The inflammation extent was graded from 0 to 3 (0, no inflammation; 1, mucosa; 2, mucosa and submucosa; 3, transmural). Crypt damage was scored as 0 to 4 (0, no damage; 1, basal 1/3 damage; 2, basal 2/3 damage; 3, crypts loss with presence of surface epithelium; 4, loss of both crypts and surface epithelium). Percentage of involvement was defined as 0 to 4 (0, 0%; 1, 1–25%; 2, 26–50%; 3, 51–75%; 4, 76–100%). The 10 data points for each mouse were averaged and colon inflammation score was expressed as means ± SEM.

### Quantitative PCR analysis of colonic cytokine expression

Two 0.5 cm segments, one from the proximal and the distal end of each colon tissue, were mixed together for RNA extraction using QIAzol Lysis Reagent. The levels of IL-6 and IL-1β mRNA were analyzed by quantitative PCR using a one-step RT-PCR kit and a Roche Light Cycler 480 Instrument. Primers sequences used in PCR amplification were as follows: IL-6 forward 5′GCTACCAAACTGGATATAATCAGGA3′ and reverse 5′CAGGTAGCTATGGTACTCCAGAA3′; IL-1β forward 5′AGTTGACGGACCCCAAAAG3′ and reverse 5′AGCTGGATGCTCTCATCAGG3′. The PCR amplification was performed by denaturing the samples at 95°C for 10 minutes, followed by 45 cycles, each cycle consisting of denaturing at 95°C for 10 seconds, annealing at 60°C for 30 seconds, and an extension at 72°C for 60 seconds. Relative mRNA expression was normalized to the expression of HPRT housekeeping gene [36].

### Statistical analysis

Data are presented as means ± SEM. Differences between groups were determined by using student's t test, one-way ANOVA followed by the least significant difference test. P values less than 0.05 were considered statistically significant.

## Results

### DNA oligos are inert and neutralize HMGB1-mediated macrophage activation

**Figure 1:**
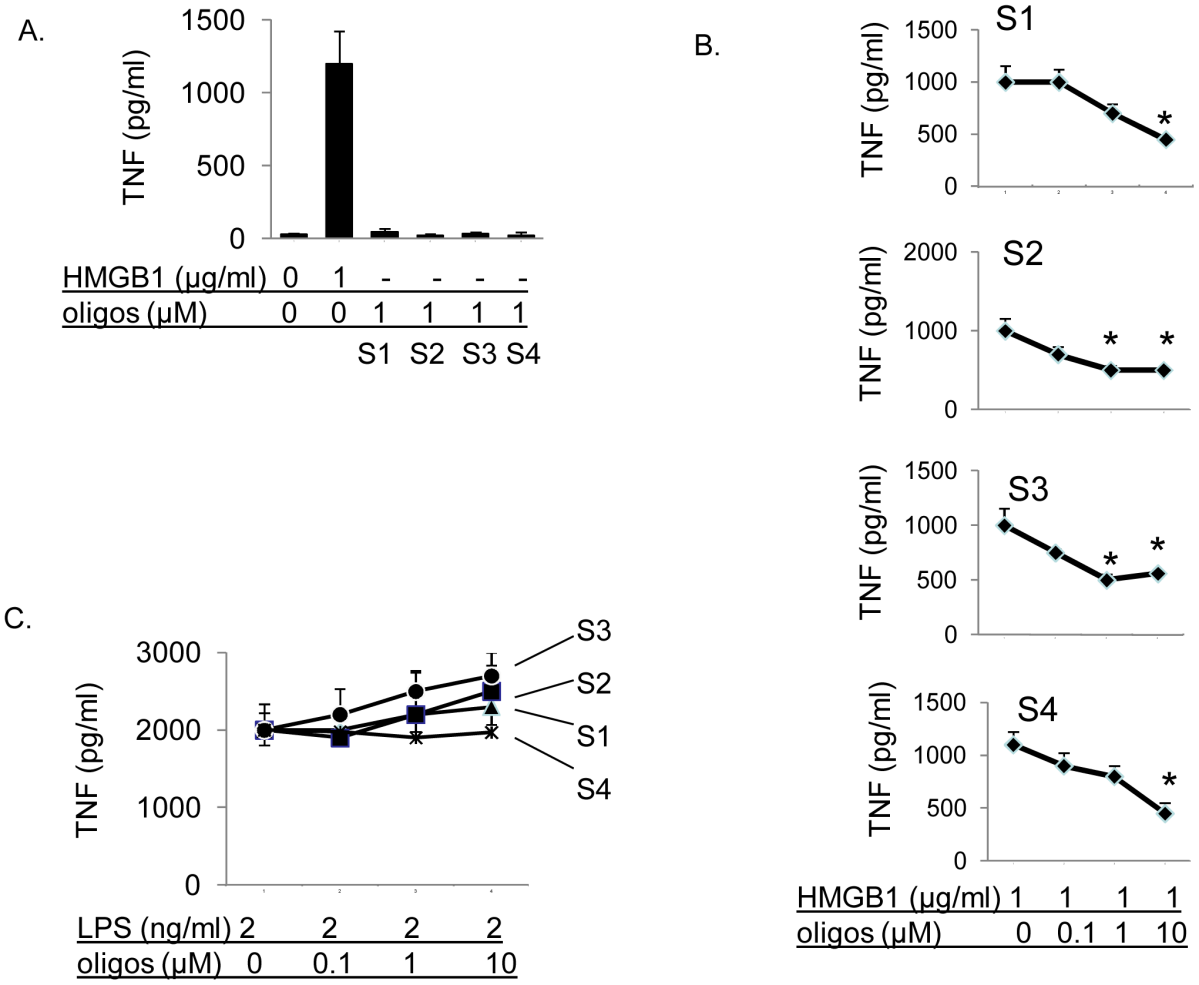
S1, S2, S3 and S4 DNA are inert and inhibit HMGB1, but not LPS-induced TNF release from macrophages. A) S1–S4 DNA is inert. Primary human macrophages were incubated with HMGB1 (positive control) or S1–S4 DNA (1 µM) for 16 hours. TNF released in the supernatants was measured by ELISA. Data are mean ± SEM from 3 experiments. B) S1–S4 DNA inhibits HMGB1-induced TNF release from cultured macrophages. Primary human macrophages in 96-well culture plates were stimulated with HMGB1 and various amounts of DNA as indicated for 16 hours. TNF released in the supernatants were measured by ELISA. N = 3 independent experiments. *: p<0.05 *vs.* HMGB1 alone. C) S1–S4 DNA does not inhibit LPS-induced TNF release. Primary human macrophages in 96-well culture plates were stimulated with LPS (2 ng/ml) plus increasing amounts of S1–S4 DNA overnight at 37°C. TNF released was measured. N = 3 experiments.

Initially we chose four DNA oligo sequences that bind to HMGB1 with high affinity [28]–[30], [38]. To determine whether DNA oligos induce inflammatory responses, we incubated human macrophages with DNA oligos S1, S2, S3 or S4 (1 µM) or with HMGB1 (1 µg/ml), and measured TNF release. None of the DNA oligos increased TNF release, suggesting that these DNA oligos themselves are inert and do not induce inflammatory responses (Fig. 1A). Next, we sought to determine whether DNA oligos can selectively neutralize HMGB1-mediated inflammatory responses. When primary human macrophages were stimulated with HMGB1 or LPS, the addition of DNA oligos S1, S2, S3 and S4 significantly inhibited HMGB1-induced, but not LPS-induced, TNF release in a concentration-dependent manner (Fig. 1B–C). To test whether free DNA oligos act synergistically with bacterial endotoxin (lipopolysaccharide, LPS) as this might occur in the gut microenvironment, we stimulated primary human macrophages with LPS in the presence of increasing concentration of DNA oligos. Notably, DNA oligos did not synergistically act with LPS to enhance LPS-induced macrophage activation (Fig. 1C). Together, these results indicate that DNA oligos specifically neutralize HMGB1-mediated macrophage activation.

### Binding of DNA beads to HMGB1

**Figure 2:**
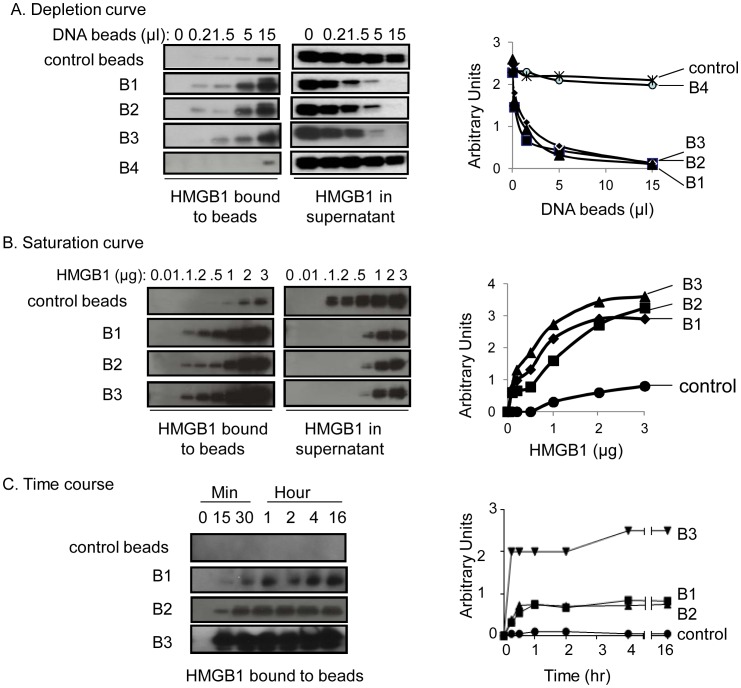
B1–B3, but not B4 beads bind to HMGB1. A) Depletion curve of HMGB1 binding to various DNA beads. Recombinant HMGB1 (2 µg per reaction) was mixed with a range of concentrations of different types of DNA beads as indicated and incubated at room temperature for two hours. The mixture was then centrifuged at 2,000 rpm for five minutes to precipitate the beads. Beads were then washed five times with PBS. Both supernatants and elute from beads were subjected to western blot analysis probed with anti-HMGB1 antibody. B) Saturation curve of HMGB1 binding to various DNA beads. Fixed amounts (20 µl drained beads) of B1–B3 or control beads were incubated with increasing amounts of HMGB1 (in 50 µl volume) at concentrations indicated for two hours at room temperature with rotation. The mixture was then centrifuged at 2,000 rpm for five minutes to precipitate the beads. The supernatants were aspirated, both supernatants and elute from beads were subjected to western blot for HMGB1 measurement using monoclonal anti-HMGB1 antibodies. The binding of 1 µg HMGB1 requires about 0.4 ng (B1 and beads) or 2.8 ng (B3) in beads, respectively. C) Time-course of HMGB1 binding to various DNA beads. B1–B3 beads (20 µl) were incubated with 500 ng of HMGB1 (50 µl total volume) at room temperature for the time periods indicated. HMGB1 bound to the beads was revealed by western blot analysis. Data are representative from 3–4 separate experiments.

Next we conjugated the oligos to sepharose beads. Beads bound to S1, S2, S3 and S4 oligos are referred as B1, B2, B3 and B4 as described in methods. To study the binding characteristics of DNA oligos to HMGB1, we incubated recombinant HMGB1 with increasing amounts of DNA beads, and analyzed bound HMGB1 by western blot analysis. B1, B2 and B3 DNA beads bind to HMGB1 (2 µg) in a concentration-dependent manner (Fig. 2A). DNA beads B1, B2 and B3 were most effective, capturing more than 90% of the HMGB1. Control beads (containing no DNA) and B4 beads (composed of kinked duplex DNA) failed to bind HMGB1, and both served as non-HMGB1 binding controls for subsequent experiments (Fig. 2A). Next, to determine the binding capacity of DNA beads, fixed amount of DNA beads (B1, B2 and B3) was incubated with increasing concentrations of HMGB1. Western blot analysis of free and bound HMGB1 revealed that DNA beads bind to HMGB1 in a concentration dependent manner with an approximate binding capacity of 1 µg HMGB1 per 50 µl DNA beads (Fig. 2B). The binding ratio of HMGB1 to DNA beads was calculated based on the maximum binding of HMGB1 to a constant amount of DNA beads; HMGB1 binds to DNA beads with approximate molar ratios of 6∶1 for B1 and B2, and 12∶1 for B3 beads. To elucidate the binding kinetics, DNA beads were incubated with HMGB1 for different times. DNA beads B1 and B2 beads bind HMGB1 by 15 min, with maximal binding observed by 30 minutes. In contrast, DNA beads B3 achieved saturation within 15 minutes (Fig. 2C). Based on these findings, we have carried out 1 hour incubation period for our subsequent binding assays to remove HMGB1 from biological samples.

### DNA beads B1 and B2 sequester different isoforms of HMGB1 but not histone protein H3 or TNF

**Figure 3:**
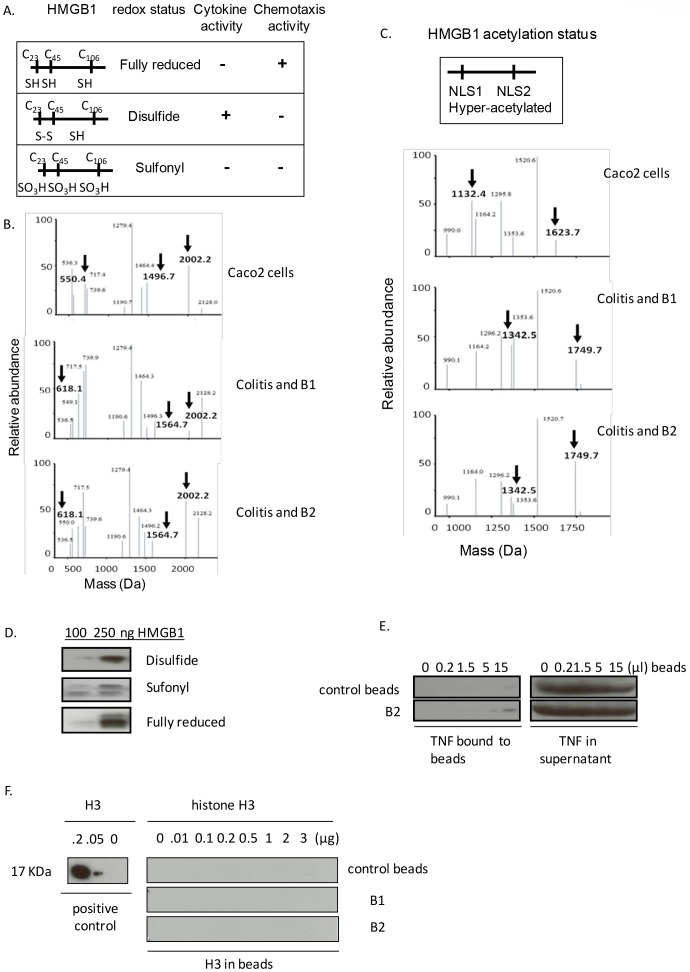
DNA beads bind to HMGB1 isoforms but not TNF or histone H3. A) Schematic representation of fully reduced, disulfide and sulfonyl forms of HMGB1 and their biological functions. B) DNA beads bind to disulfide HMGB1 in feces from colitis mice. The redox state of HMGB1 captured by DNA beads B1 or B2 from feces of colitis mice was assessed by trypsin digestion followed by LC/MS analysis. Cell lysate from colorectal epithelial (Caco 2) cells was used as control for intracellular HMGB1. Diagnostic peptides showed with the presence of molecular weights 1496 and 550 (Da) indicates reduced cysteines 23 and 45 respectively, whereas the appearance of molecular weights of 1564 and 618 (Da) indicates forming of an intramolecular disulfide linkage between cysteines 23 and 45 (arrows). Cys 106 (MW = 2002) is in reduced form in all samples presented here. Data are representative from two separate experiments. C) DNA beads bind to hyper-acetylated form of HMGB1 in feces from colitis mice. Representative spetra of the LC-MS traces of the above samples digested with endopeptidase GluC. The presence of peptides with molecular weights 1624 and 1132 Da (arrows) indicates the hypo-acetylation of lysine residues in NLS1 and NLS2 regions respectively; whereas the presence of peptides of 1749 and 1342 Da (arrows) indicates hyper-acetylation of lysines within NLS1 and NLS2 regions. Data are representative from two separate experiments. D) B2 beads bind to redox modified HMGB1 proteins. Increasing amounts of various HMGB1 proteins (100 or 250 ng) were added to B2 beads (20 µl) and the mixture (50 µl total volume) was incubated at room temperature for two hours. The mixture was then centrifuged and HMGB1 bound to beads were revealed by western blotting with anti-HMGB1 antibodies. N = 3 experiments. E) B2 beads do not bind to TNF. To examine the specificity of DNA beads and HMGB1 binding, human TNF (200 ng) was incubated with increasing amounts of B2 beads (as indicated) at room temperature for two hours. After incubation, TNF remaining in the supernatants and bound to the beads were analyzed by western blot probed with anti-human TNF antibodies. Data are representative from three separate experiments. F) B2 beads do not bind to human histone H3. B2, B1 or control beads (5 µl) in each tube were incubated with increasing amounts of human histone H3 (0, 0.01, 0.1, 0.2, 0.5, 1, 2, 3 µg) for two hours at room temperature. The amount of histone H3 bound to DNA beads was analyzed by western blot using anti-histone H3 antibodies. Histone H3 protein was used as a positive control in western blot. Data are representative of 3 repeats.

HMGB1 contains three conserved redox-sensitive cysteines (C23, C45 and C106) that determine the biological activity of extracellular HMGB1 [19]–[22] (Fig. 3A). In this context, the fully reduced form of HMGB1 dominates intracellular HMGB1 pool, whereas macrophage activation markedly induces the formation of HMGB1 bearing disulfide bonds between Cys23 and Cys45 [24]. It is theoretically possible that in IBD, cell death and activation of immune cells may contribute to accumulation of different isoforms of HMGB1. Specifically, cell death by necrosis may lead to passive release of fully reduced HMGB1, whereas cell activation may result in active release of disulfide HMGB1. To test this hypothesis, HMGB1 was sequestered from colitis feces using DNA beads. HMGB1 captured by DNA beads was further subjected to liquid chromatography and mass spectrometric analysis (LC-MS/MS) to assess redox form and acetylation status as described previously [24], [25]. As expected, HMGB1 captured by B1 or B2 beads from feces of colitis mice contained a mixture of pro-inflammatory isoforms (cytokine-inducing disulfide HMGB1 and fully reduced HMGB1). As control, we also analyzed HMGB1 extracted from cultured human colorectal epithelial adenocarcinoma Caco-2 cell line, confirming the fully reduced intracellular form of HMGB1 (Fig. 3B). It has been established that HMGB1 released by cell activation is hyper-acetylated. In contrast, HMGB1 released by cell death is hypo-acetylated [23], [24]. HMGB1 from colitis feces and captured by DNA beads was hyper-acetylated in both nuclear localization sequences (NLS1 and NLS2) (Fig. 3C). In contrast, HMGB1 isolated from Caco 2 cells was hypo-acetylated, implicating the active and passive release of HMGB1 respectively. Next, to confirm the binding of DNA beads to different isoforms of HMGB1, we generated different redox forms of HMGB1 by exposing HMGB1 to either hydrogen peroxide or dithiothretol as described previously [19] prior to incubation with DNA beads. B1 and B2 beads bind to all three redox-modified HMGB1 proteins in a concentration-dependent manner (Fig. 3D). Taken together, B1 and B2 beads can sequester different redox and acetylated forms of HMGB1 found in the fecal environment of colitis animals. It is also theoretically plausible that DNA beads may exhibit non-specific interaction with either other DNA binding proteins including histones or other inflammatory mediators present in fecal samples. To elucidate this possibility, we incubated DNA beads (B1, B2 and control beads) with TNF or histone H3 (one of the core histones). Western blot analysis of free and bound protein revealed that both control and DNA beads failed to bind to significant amounts of either TNF or histone H3 (Fig. 3E and 3F).

### DNA beads do not affect cell viability

**Figure 4:**
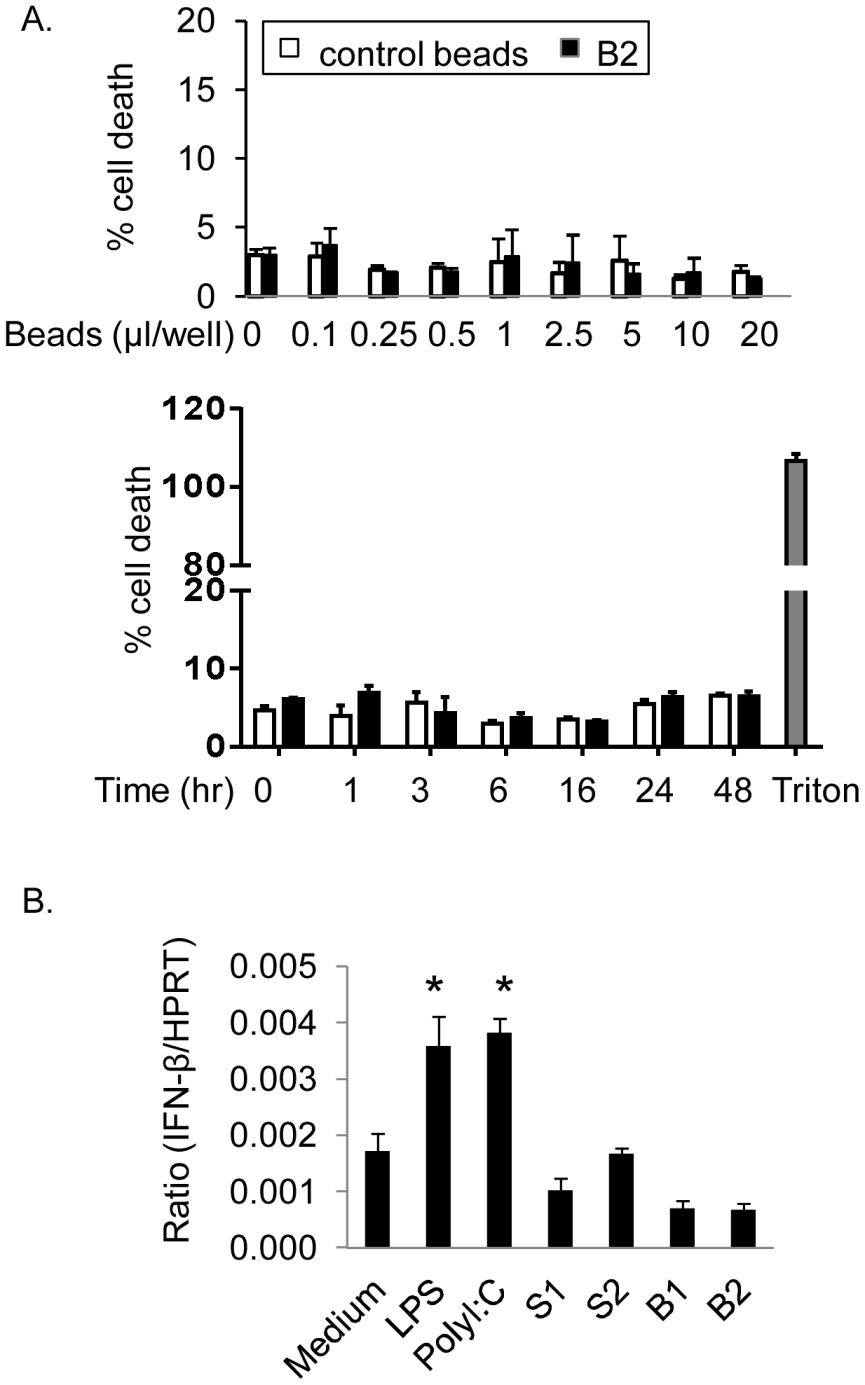
DNA beads are neither cytotoxic nor able to induce type I IFN expression. A) B2 DNA beads are not cytotoxic. Caco-2 (human epithelial colorectal adenocarcinoma) cells in 24-well plates were incubated with increasing amounts of B2 or control beads overnight at 37°C in Opti-MEM medium. In time course experiments, fixed amount of B2 or control beads (5 µl) were incubated with cells for the time periods indicated. Supernatants were collected and LDH levels were analyzed by cytotoxicity kit. Triton X-100 (2%) was used as a positive control. Data are means ± SEM, N = 3 experiments. B) DNA beads do not induce type I IFN expression in macrophages. RAW 264.7 cells were incubated with LPS (2 ng/ml) or poly I∶C (1 µM, positive controls), S1, S2 or B1, B2 beads for 24 hours at 37°C. Cell total RNA was extracted; the expression IFN-β and HPRT mRNA was analyzed by quantitative PCR and presented as the ratio of IFN-β/HPRT. N = 3–4 per group. *: p<0.05 vs. medium alone.

We have demonstrated that S1, S2, S3 and S4 oligos selectively neutralize HMGB1 mediated inflammatory responses (Fig. 1B). It is theoretically plausible that incubation with DNA beads may induce cell toxicity resulting in reduced inflammatory responses. To investigate this possibility, Caco-2 cells or Hela (human cervical cancer cell line) cells were cultured with B2 beads. Analysis of secreted cell death marker, lactate dehydrogenase (LDH), demonstrated that LDH levels were not significantly different between Caco-2 cells exposed to different amounts of B2 or control beads for increasing incubation periods compared with medium alone (Fig. 4A). The non-toxic effects of beads were further confirmed by exposing Hela cells to B2 beads (data not shown).

### Neutralizing effects of DNA beads are independent of type 1 interferon (IFN) expression

Previous studies showed that increased expression of type 1 IFN, induced by stimulation with self or non-self DNA, protects mice from experimental colitis [39]. Type 1 IFN has been evaluated in pilot clinical trials for the treatment of active ulcerative colitis [40], [41]. Based on these studies, it is possible that the inhibitory effects of DNA beads are mediated via the production of Type I IFN. To test this possibility, we measured type 1 IFN mRNA levels in the presence of oligos or DNA beads using quantitative PCR. Oligos (S1 and S2) and DNA beads (B1 and B2 beads) failed to induce type I IFN mRNA expression in RAW 264.7 cells, whereas LPS or poly I∶C induced IFN expression (positive controls) (Fig. 4B). These results further confirm previous observations that the DNA oligos used in these studies are inert and do not induce inflammatory phenotype in macrophage population [30].

### DNA beads are stable in various biological conditions

**Figure 5:**
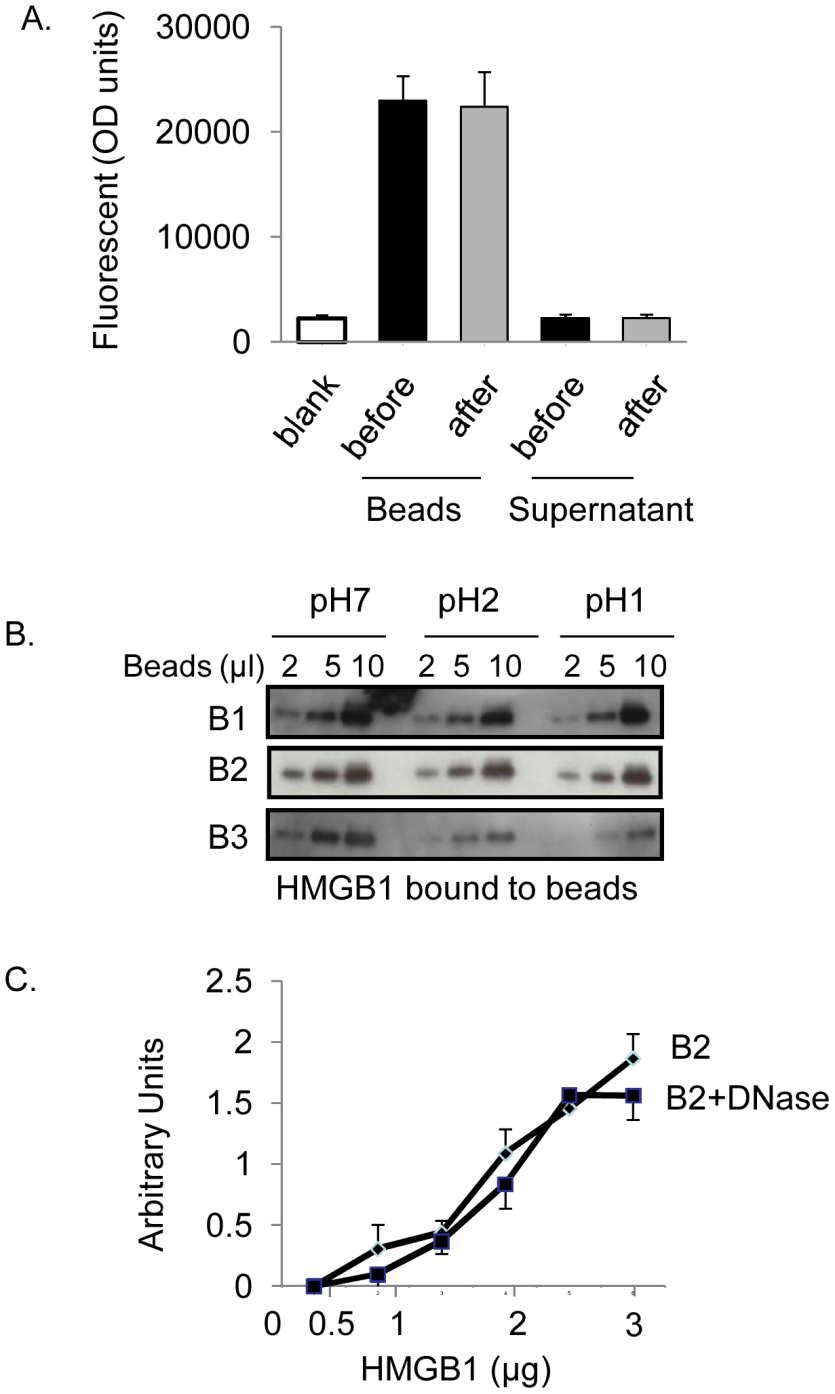
DNA beads are stable. A) B2 DNA beads are stable in fecal matters. Fluorescent FAM-labeled B2 beads (50 µl) was incubated with fecal extract of colitis mice (300 µl) for 2 hours at 37°C. After centrifugation, beads were washed five times with PBS and re-suspended as 50% slurry. The fluorescence intensity in both supernatants and beads (before and after the incubation) was measured by using a microplate spectrophotometer at excitation of 494 nm. N = 3 separate experiments. B) B1 and B2 beads are resistant to acidic pH, B3 is not. B1, B2 or B3 beads at the amounts indicated were incubated with HMGB1 (500 ng) in either solutions of PBS (pH 7) or HCl (pH 2 or 1) at room temperature for 2 hours. HMGB1 bound to the beads was revealed by western blot analysis. Data are representative from 3 separate experiments. C) B2 beads are resistant to degradation by DNase I. DNA beads were incubated with DNase I (40 ng/ml, a concentration observed in biological fluid [35]) for 30 minutes at 37°C. The binding of HMGB1 by B2 beads with or without DNase I treatment was analyzed by western blot. Data are from 3 separate experiments.

To examine the stability of DNA-coated sepharose beads in biological fluid at body temperature, we used fluorescent FAM-labeled DNA coated beads. The FAM-labeled DNA beads were incubated with fecal extract at 37°C for two hours and the amounts of free DNA in the supernatant were evaluated. There were no significant differences in the amount of fluorescent DNA released in the supernatant between beads exposed to fecal extract or not (Fig. 5A) [42]. Next, to evaluate whether HMGB1 binding ability of DNA beads remain intact under different pH conditions, DNA beads were incubated with HMGB1 at neutral or acidic pH; all three beads (B1, B2 and B3) efficiently captured HMGB1 from solutions in a concentration-dependent manner. Notably, only B1 and B2 beads, but not B3 beads, retain the binding capacity at pH 1 or 2 (Fig. 5B). This feature is an advantage as DNA beads will be administered orally in colitis mice and exposed to the acidic environment of the stomach. In addition, we determined whether DNA bound to the beads is susceptible to DNAse I mediated digestion. B2 beads were incubated with physiological concentration of DNase I (40 ng/ml) [35] prior to incubation with recombinant HMGB1. Pre-exposure to DNase I did not alter the binding efficiency of B2 beads to HMGB1 suggesting that bound DNA is resistant to DNase I; presumably protected by their phosphorothioated backbones (Fig. 5C).

### Intraluminal HMGB1 induces tissue damage

**Figure 6:**
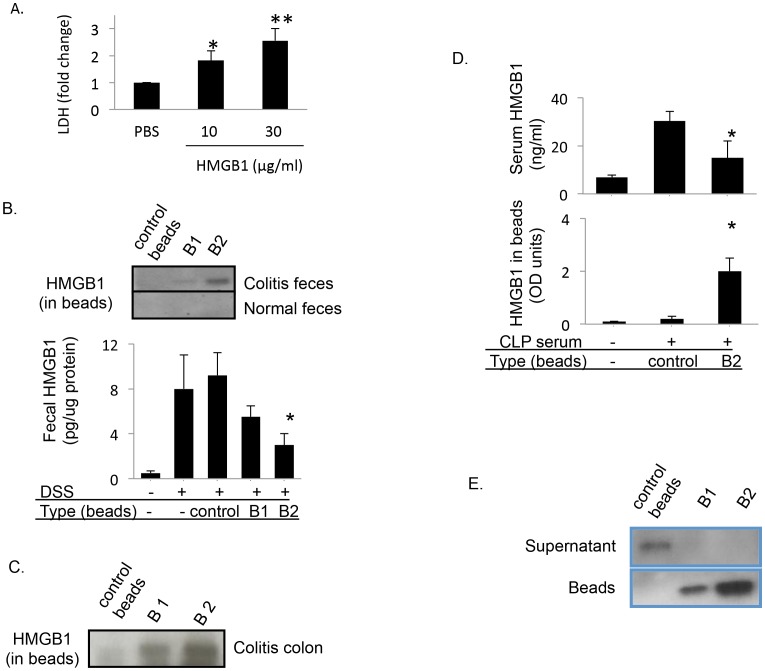
HMGB1 causes increased intestinal LDH release; and DNA beads capture HMGB1 in biological fluids *ex vivo*. A) HMGB1 (10 and 30 µg/ml) or PBS (all in 500 µl volume) were injected into freshly isolated full length colons from mice and incubated at room temperature for 1 hour. Colonic lavage was collected and LDH levels were measured. N = 3 preparations per group. *: p<0.05, **: p<0.01 *vs.* PBS group. B) DNA beads capture HMGB1 from feces of colitis mice induced by DSS. Feces in the colon of colitis mice were gently flushed out with cold PBS containing 1× protease inhibitor, and the suspension was rotated for an hour at room temperature in the presence of gentamycin and imipenum. After centrifugation to remove fecal debris, fecal extract containing 40 µg of total protein was incubated with B1, B2 or control beads at room temperature for an hour. After washing with PBS, beads were boiled and eluate was analyzed by western blot probed with anti-HMGB1 antibodies. N = 3–4 mice per group. *: p<0.05 *vs.* DSS control. C) Female BALB/c mice (8–12 weeks old, n = 10–12 per group) were given 4% dextran sodium sulfate (DSS) dissolved in drinking water ad libitum for five days, and then switched to normal water for three days. DNA beads capture HMGB1 from inflamed colon from colitis mice. Mice were euthanized on day 9th after overnight fasting. Both B1 and B2 beads captured HMGB1 from colon culture of colitis mice (see Methods) compared to control beads. D) DNA beads remove HMGB1 from septic mice sera. Serum (20 µl) from normal or septic mice was incubated with 50 µl of B2 or control beads at room temperature for an hour. Samples were then centrifuged at room temperature for five minutes. The binding of DNA beads with HMGB1 was measured using western blot or ELISA. Data shown are means ± SEM from 3–5 mice per group. *: p<0.05 *vs.* control beads. E) DNA beads capture HMGB1 from cell supernatants. RAW 264.7 cells in 6-well plate were stimulated with LPS (100 ng/ml) for 16 hours, and HMGB1 containing supernatant (1 ml) was collected and concentrated 10× through centrifugation with Microcon centrifugal filters. The concentrated RAW 264.7 cell supernatant was then incubated with beads (20 µl) containing control, B1 or B2 beads at room temperature for an hour with rotation. HMGB1 content in both supernatant and beads were measured by western blot. N = 2 repeats each performed in duplicate.

Analysis of the focal concentration of HMGB1 in the colon have demonstrated that HMGB1 is released by colonic tissue in animal models of colitis [14], [17], [18]. Based on our previous findings that HMGB1 increases the permeability of human epithelial cells and that neutralization of HMGB1 ameliorates gut barrier dysfunction [15], [16], we hypothesized that intralumenal HMGB1 might mediate intestinal epithelial dysfunction and contribute to intestinal inflammation. To test this hypothesis, we infused freshly isolated mouse colon segments with HMGB1 or vehicle PBS. HMGB1 infused in the colon resulted in a 2–3 fold increase in LDH release, a marker of epithelial damage, as compared to PBS controls (Fig. 6A), indicating that intraluminal presence of HMGB1 acts as a mediator of epithelial damage.

### DNA beads bind and remove HMGB1 from inflamed colon and feces

HMGB1 levels are increased in inflamed colons and in feces with both human and murine colitis [13], [14], [17], [18]. To demonstrate that the fecal micro-environment did not interfere with binding of HMGB1 to DNA beads, we first analyzed HMGB1 captured by B2 beads in the presence and absence of fecal debris. BALB/c mice were subjected to DSS to induce colitis as described in Methods. B2 beads (20 µl) were added directly to fecal matters obtained from these colitis mice. B1 and B2 beads (20 µl) captured HMGB1 from the feces extracts from colitis mice whereas control beads did not (Fig. 6B). Next, we performed an *ex vivo* colon cultures with colons isolated from mice subjected to DSS-induced colitis. B1, B2 or control beads were infused in the colons isolated from DSS-induced colitis mice. B1 and B2 beads captured significant amounts of HMGB1 from *ex vivo* colon culture compared to control beads (Fig. 6C). Taken together, these observations demonstrate that HMGB1 levels are elevated locally in the colonic micro-environment, and DNA beads can be utilized to sequester and reduce HMGB1 levels from colitis colon.

### DNA beads bind and remove HMGB1 from septic mouse serum

Circulating HMGB1 levels are elevated in many inflammatory disease conditions including sepsis [10], [11], [43], [44].To test whether DNA beads capture and remove HMGB1 in the serum, we utilized an established model of murine sepsis induced by cecal ligation and puncture (CLP) [11], [45]. In this model, a surgically created diverticulum of the cecum is punctured. This procedure reproducibly results in elevated serum HMGB1 levels compared to normal controls (Fig. 6D) [11]. When serum samples containing high levels of HMGB1 were incubated with B2 or control beads (20 µl), B2 beads but not the control beads sequestered significant amount of HMGB1 from septic serum. In agreement with these findings, HMGB1 bound to the B2 beads was significantly higher than that bound to the control beads (Fig. 6D), suggesting that DNA beads are capable of removing HMGB1 from biological samples *ex vivo*.

### DNA beads sequester HMGB1 *ex vivo* from activated cell supernatants

Next we investigated whether DNA beads can efficiently sequester HMGB1 from biological samples. RAW 264.7 cells were stimulated with LPS to induce release of HMGB1 in cell supernatant [10]. B1 and B2 beads efficiently bound and removed HMGB1 from the activated RAW cell supernatants while control beads did not (Fig. 6E). B3 beads failed to bind HMGB1 in cell supernatants and were eliminated from further *in vivo* studies. As B2 beads consistently showed greater binding to HMGB1 than the other DNA constructs in *ex vivo* studies, it was chosen for the subsequent *in vivo* studies.

### DNA beads B2 ameliorates inflammation in animal models of acute and chronic colitis

**Figure 7:**
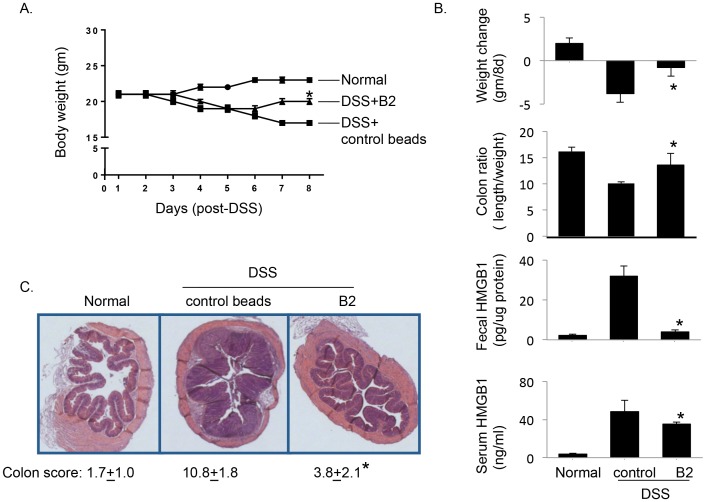
Administration of B2 beads ameliorates inflammation in DSS-induced colitis in mice. Female BALB/c mice (10 mice per group) were given 4% DSS in drinking water to induce colitis water for 5 days to induce colitis, and then switched to normal water. Mice were orally administrated with 300 µl (50% slurry, gavage) of B2 or control beads on days 0, 2, 4 and 6 after DSS initiation and were euthanized on day 8. A) Treatment with B2 beads reduced body weight loss in DSS-induced colitis mice. *: p<0.05 *vs.* control beads group. B) Body weight change, colon measurements (weight and length) and levels of serum and fecal HMGB1 in colitis mice treated with B2 or control beads. *: p<0.05 *vs.* control beads group. C) Histological evaluation of B2 or control beads treated colitis mice. Representative H&E staining of colons from normal, control or B2 beads-treated colitis mice is shown. Histological scores of colons are shown. N = 10 mice per group. *: p<0.05 *vs.* control beads group. Magnification: ×40.

Reasoning that administration of DNA beads might ameliorate inflammation in colitis conditions; we examined the effects of DNA beads in models of both acute (DSS-induced colitis) and chronic colitis (IL-10 KO mice that spontaneously developed colitis [46]). Oral administration of B2 beads to mice with DSS-induced colitis significantly improved weight loss compared to animals receiving control beads (weight change = −0.8±0.4 gm/8 days in B2 beads group and −3.8±0.5 gm/8 days in control beads groups, respectively. *p<0.05. Fig. 7A–B). We observed that animals receiving B2 beads also had significantly lower levels of circulating and fecal HMGB1 (Fig. 7B), decreased colonic wall thickening as revealed by less tissue swelling, recovery of crypt structure (Fig. 7C), and significantly improved histological scores for the colon compared to control beads-treated group (scores = 3.8±2.1 in B2 beads group *vs.* 10.8±1.8 in control beads groups. p<0.05. N = 10 per group. Fig. 7B–C). To confirm the specificity of HMGB1-binding DNA beads in mediating improvement of colitis, we administered B4 beads as control beads to DSS-colitis mice. Our prior analysis has demonstrated that B4 beads failed to sequester HMGB1 (Fig. 2A). As expected, administration of B4 beads did not improve DSS-induced weight loss in mice (the body weight change = −3.0±0.4 in B4 beads group vs. −2.6±0.3 gram/8 days in control beads group; N = 9–10 mice/group. P = N.S.), and did not reduce circulating levels of HMGB1 (HMGB1 = 56±4 in B4 beads group *vs.* 50±2 ng/ml in control beads group. N = 9–10 mice per group, p = N.S.).

**Figure 8:**
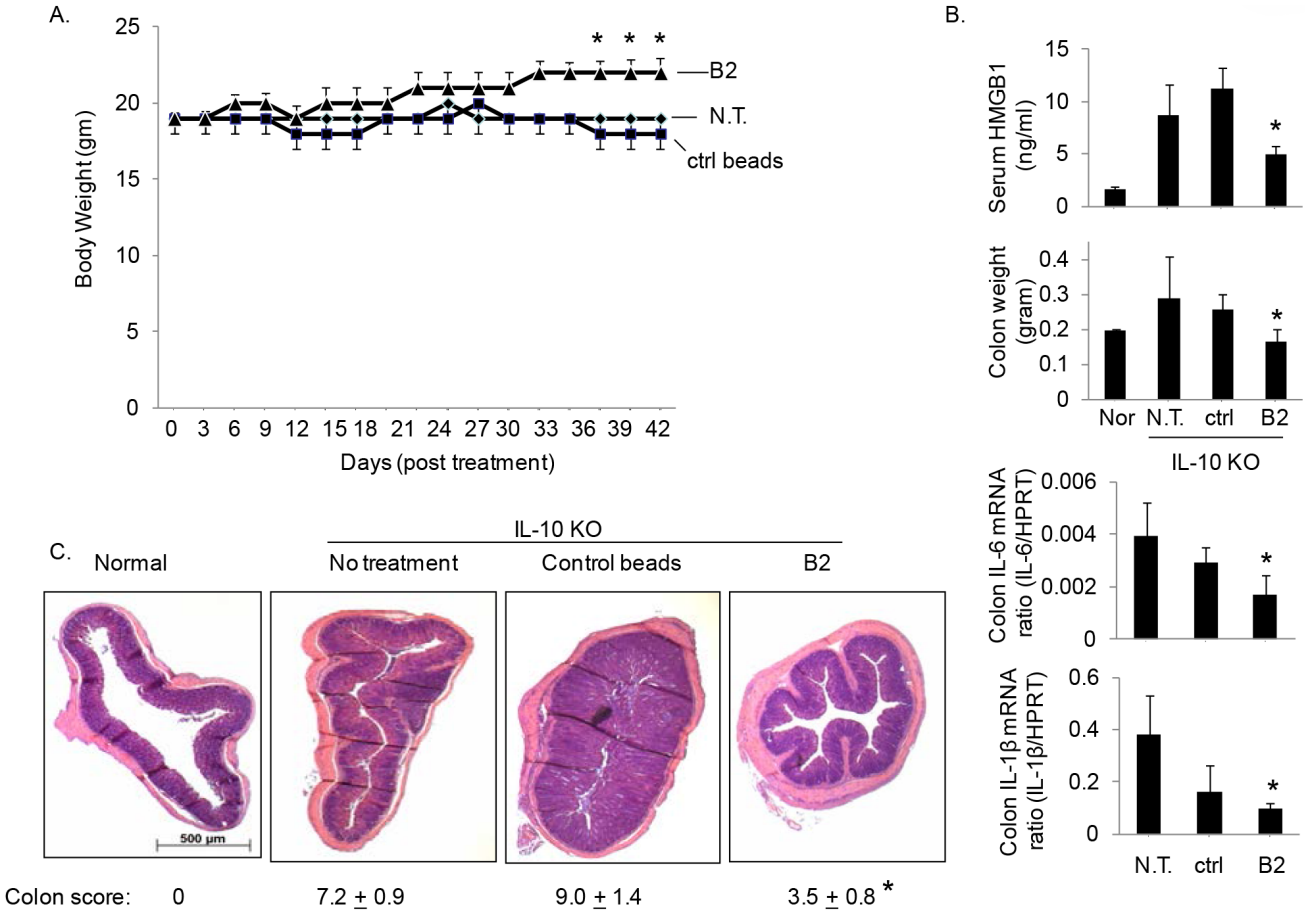
Administration of B2 beads ameliorates colitis-induced inflammation in IL-10 KO mice. Twelve weeks old female IL-10 KO mice were orally administrated with 300 µl (50% slurry, gavage) of B2 or control beads three times a week for a total of six weeks. A) Treatment with B2 beads increased body weight in mice. *: p<0.05 *vs.* control beads group. N = 5–7 mice per group. N.T. = no treatment. B) Colon and serum measurements of IL-10 KO mice treated with B2 or control beads. Colon measurements (weight, expression of IL-6 and IL-1β mRNA) and serum HMGB1 levels in IL-10 KO mice treated with B2 or control beads. *: p<0.05 *vs.* control beads group. N = 5–7 mice per group. N.T. = no treatment. C) Histological evaluation of B2 or control beads-treated IL-10 KO mice. Representative H&E staining of colon from wild type (C57BL/6) or IL-10 KO mice and histological scores (see Methods) of colons are shown. *: p<0.05 *vs.* control beads group. N = 5–7 mice per group. Magnification: ×40.

In agreement with the findings in DSS-induced colitis model, administration of B2 beads significantly improved disease pathogenesis in IL-10 knockout (KO) mice with spontaneously developed colitis. Oral administration of B2 beads significantly increased body weight compared to control beads or unmanipulated group after 6 weeks of treatment (final body weight = 23±2 grams in B2 beads group, 19±2 or 18±3 grams in untreated or control beads groups respectively. N = 5 or 7 mice per group, *p<0.05 B2 beads *vs.* control beads group, Fig. 8A). Consistent with these findings, the IL-10 KO mice receiving B2 beads exhibited a significant reduction in serum HMGB1 levels, and lower levels of colonic IL-6 and IL-1β expression as compared to control beads group (Fig. 8B). These changes were also reflected in the severity of colon injury. Gross inspection of the colon revealed decreased swelling in mice treated with B2 beads, as compared to mice treated with control beads. Histological evaluation demonstrated severe thickening of the colon walls in groups of untreated or control beads-treated mice; significantly less thickening was observed with administration of B2 beads (histological score = 3.5±0.8 in B2 beads group, and 7.2±0.9 or 9.0±1.4 in untreated or control beads treated groups respectively. p<0.05, B2 beads vs. control beads groups. Fig. 8C). Taken together, HMGB1 plays an important role in mediating disease severity in colitis, and sequestering HMGB1 using HMGB1-binding DNA beads significantly attenuate disease pathogenesis in animal models of colitis.

## Discussion

Together, these findings establish that the DNA beads can selectively sequester HMGB1 from colitis colons and improve disease pathophysiology in experimental models of colitis. Initially identified as a nucleosomal protein, cytokine inducing activities of HMGB1 are now well established. HMGB1 is secreted by many cell types and specifically stimulates synthesis of proinflammatory cytokines by monocytes and macrophages [12], [32], [47], [48]. It acts as a necessary and sufficient mediator of sepsis pathophysiology [12], [49], [50]. Neutralization of HMGB1 using anti-HMGB1 antibodies or HMGB1-specific antagonists have been successfully ameliorated disease severity in many experimental disease models [11], [32], [47]. Ample evidence indicates that HMGB1 is an important mediator in the pathogenesis of colitis and colitis associated carcinoma [13], [17]. Increased circulating levels of HMGB1 have been observed in animal models of IBD and are positively associated with disease severity [13]. Fecal HMGB1 is a novel marker of intestinal mucosal inflammation with important implications in the pathogenesis of human IBD [51]. HMGB1 is secreted by inflamed human intestinal tissues, and abundantly found in stools of IBD patients [14], [18]. It is established that high levels of HMGB1 increase epithelial cell permeability and impair intestinal barrier function [15]. Together these studies suggest that HMGB1 is an important target in IBD, and sequestration and or neutralization of HMGB1 would be beneficial for controlling the disease severity in colitis. Here we utilized the strong DNA binding property of HMGB1, and developed DNA beads that selectively bind and sequester HMGB1 from biological fluids.

Recent studies emphasize that during tissue inflammation and repair, HMGB1 is present in different redox states in the extracellular environment. The redox states of the three conserved cysteine residues within HMGB1 regulate its receptor-binding ability and subsequent biological outcome [12], [32], [47]. Given the importance of presence of various redox forms of HMGB1, we assessed the potential binding of DNA beads to different isoforms of HMGB1. Our *in vitro* assessments of redox-modified recombinant HMGB1 showed that DNA beads dose dependently sequester different isoforms of HMGB1. An interesting question is whether any specific redox isoform of HMGB1 is predominantly present during active phase of colitis. To this end, we have analyzed the redox status of HMGB1 sequestered from colitis fecal material using DNA beads. As expected, fecal HMGB1 captured by DNA beads contained a mixture of redox isoforms including cytokine-inducing disulfide HMGB1 and chemotaxis-inducing fully reduced HMGB1. We found that the predominant form of HMGB1 is the disulfide isoform suggesting a role of HMGB1 in mediating and perpetuating mucosal inflammation. It is noteworthy that HMGB1 specific receptors, TLR4 and RAGE, are up-regulated in colon tissues in colitis environment [18], implicating the importance of identifying a treatment strategy that will effectively sequester and neutralize different isoforms of HMGB1 in IBD.

The objective of this study was to develop HMGB1 specific DNA beads that could abrogate disease in murine models acute and chronic colitis, and thus have a potential as a therapy for human IBD. We have now demonstrated that administration of DNA beads significantly ameliorated the disease severity associated with improvement in clinical measures of colitis, including body weight loss; morphological parameters such as colon weight; inflammatory markers, and histological scores for colonic tissue injury. We did not observe toxicity in animals treated with either DNA beads or the control beads, and we found that the neutralizing effect of DNA beads is independent of type 1 IFN expression. Another important finding in our study is that DNA beads bind specifically to HMGB1, and not to other proteins, including DNA binding protein histone H3. Although histone H3 (one of the core histones and a DAMP prototype) failed to bind to DNA beads in our experiments, which may attribute to the requirement of specific sequence and length of (146 bp for core histones) of DNA for binding to histones [52], we cannot exclude the possibility that other proteins may also bind to DNA beads. DNA beads may bind to HMGB2 and HMGB3 [9], histones H1 and B4 and hyaluronic acid. However, none of these DNA binding proteins are present in significant amounts in the gastrointestinal tract.

In summary, here we describe a novel approach using HMGB1-specific DNA beads to effectively sequester HMGB1, and improve disease pathogenesis in clinically relevant models of colitis. Given the high affinity of HMGB1 to DNA, this DNA-based HMGB1 sequestration therapy in IBD may provide therapeutic advantages.
